# Insights into safety and efficacy of positioning strategies in percutaneous nephrolithotomy

**DOI:** 10.1097/JS9.0000000000003375

**Published:** 2025-09-10

**Authors:** Yawei Zhang, Hongjin Shi, Haifeng Wang

**Affiliations:** Department of Urology, The Second Affiliated Hospital of Kunming Medical University, Kunming, Yunnan, China

*Dear Editor,*

Percutaneous nephrolithotomy (PCNL) remains the preferred intervention for renal calculi exceeding 20 mm in diameter, particularly staghorn stones^[1]^. Conventional prone positioning during PCNL substantially compromises cardiopulmonary function and hemodynamic stability. To mitigate anesthetic risks, reduce respiratory–circulatory complications, and optimize surgical maneuverability, novel operative positions continue to be explored. A recent systematic review and network meta-analysis by Li et al^[2]^ evaluated five PCNL positions – supine, oblique supine, flank, split-leg oblique supine/flank, and prone – accounting for the surgeon’s learning curve. Their findings confirmed the safety and efficacy of all approaches. The supine and oblique supine positions demonstrated advantages over prone positioning through higher stone-free rates (SFR) and fewer complications. Furthermore, the split-leg position emerged as a potentially superior option due to improved SFR, reduced operative duration, shorter hospital stays, and diminished hemoglobin loss.

This comprehensive analysis offers significant merits and valuable insights. For example, only randomized controlled trials (RCTs) were included, which makes the results more reliable. Additionally, the application of network meta-analysis enabled both direct comparisons among diverse PCNL positions and indirect assessments of interventions lacking head-to-head clinical trial data. Nevertheless, we respectfully suggest several points for discussion to augment the quality of such systematic reviews and meta-analyses. During the preparation of this work the authors didn’t use the AI and AI-assisted technologies^[3]^.

First, there is an inconsistency between the main text and Table 1 regarding the number of studies analyzing operative time. Although the text states that comparison of operative time was generated from 15 RCTs, Table 1 only documents 14 entries. This discrepancy likely arises because the study by Falahatkar et al^[4]^, which compared operative times, was not reflected under the “Outcome” section in the table. A similar issue arises in the comparison of hospital stay. Aligning textual descriptions with tabular data would strengthen methodological rigor.

Second, tract formation time represents a critical parameter for surgeons. Although four included studies examined this metric, variations in compared positions precluded meta-analysis. Given that the authors are based in China, inclusion of Chinese-language articles could have provided unique data on tract formation time, potentially enabling meta-analysis for this specific outcome.

Finally, except for Desoky E’s investigation^[5]^, enrolled participants were exclusively adults with BMIs exceeding the healthy threshold of 24. This demographic focus suggests the findings are particularly applicable to preoperative counseling for obese or overweight patients rather than the general population.

In conclusion, we commend the authors for delivering a thorough synthesis that advances evidence-based position selection in PCNL. Given the current scarcity of RCTs, future well-designed studies minimizing bias are warranted to further validate the split-leg position as a potentially optimal strategy.

## Data Availability

Not applicable.

## References

[1] Geraghty RM Davis NF Tzelves L. Best practice in interventional management of urolithiasis: an update from the european association of urology guidelines panel for urolithiasis 2022. Eur Urol Focus 2023;9:199–208. 35927160 10.1016/j.euf.2022.06.014

[2] Li P Ma Y Liao B. Comparison of safety and efficacy of different positions in percutaneous nephrolithotomy: a network meta-analysis. Int J Surg 2024;110:2411–20. 38445503 10.1097/JS9.0000000000001130 PMC11020106

[3] Agha RA Mathew G Rashid R. Transparency in the reporting of Artificial INtelligence – the TITAN guideline. Premier J Sci 2025;10:100082.

[4] Falahatkar S Moghaddam AA Salehi M Nikpour S Esmaili F Khaki N. Complete supine percutaneous nephrolithotripsy comparison with the prone standard technique. J Endourol 2008;22:2513–17. 19046091 10.1089/end.2008.0463

[5] Desoky EAE Sakr AM ElSayed ER Ali MM. Ultra-mini-percutaneous nephrolithotomy in flank-free modified supine position vs prone position in treatment of pediatric renal pelvic and lower caliceal stones. J Endourol 2022;36:610–14. 34861776 10.1089/end.2021.0557

